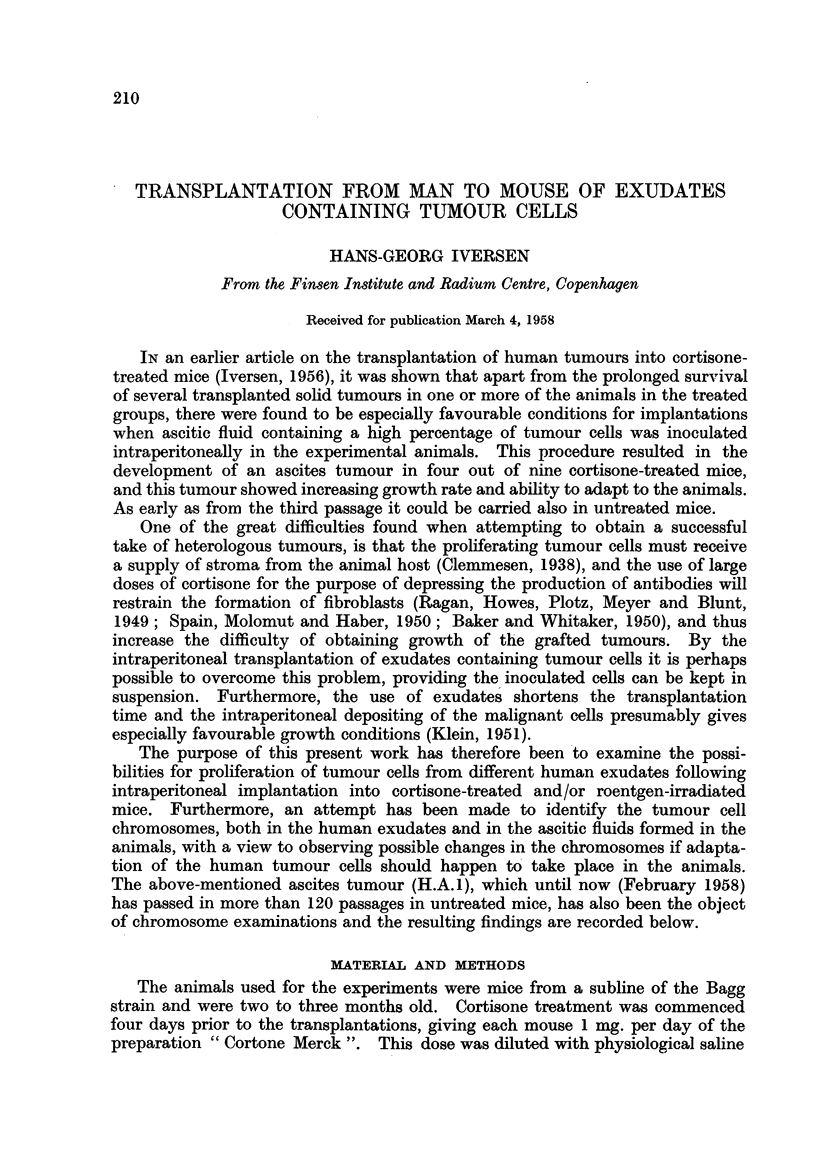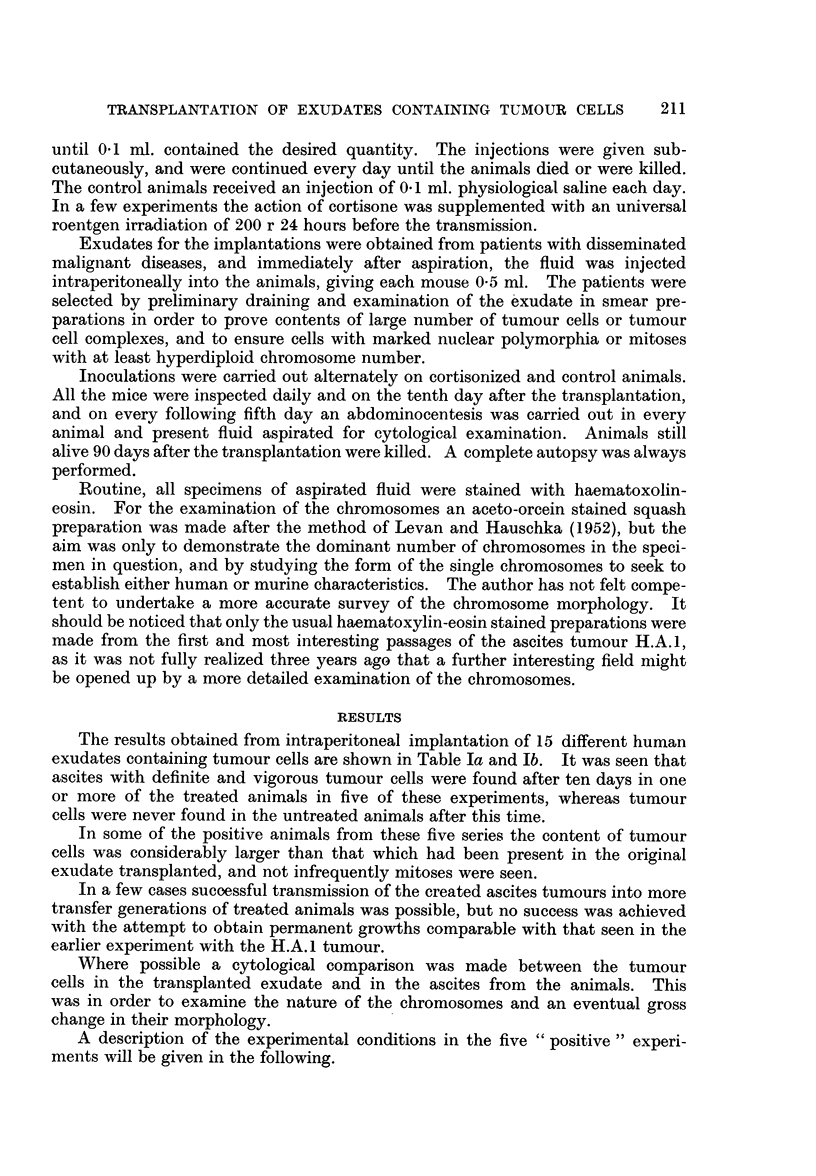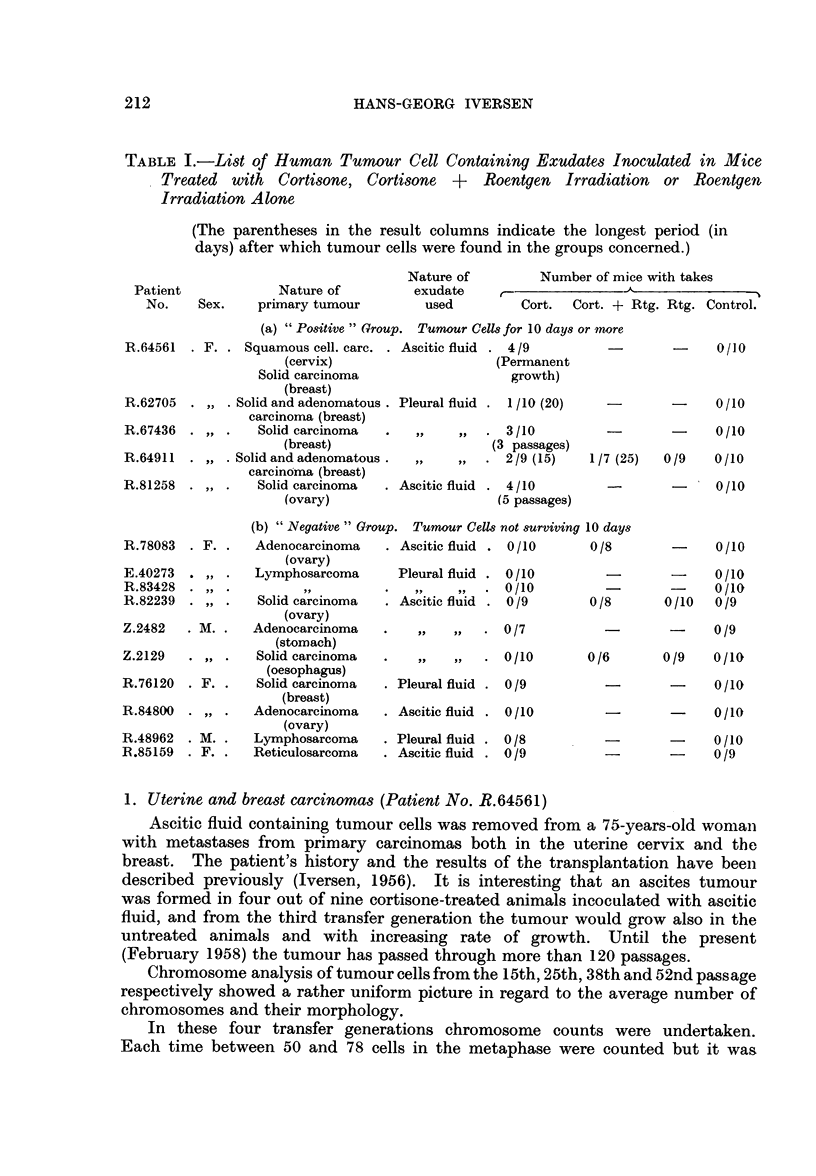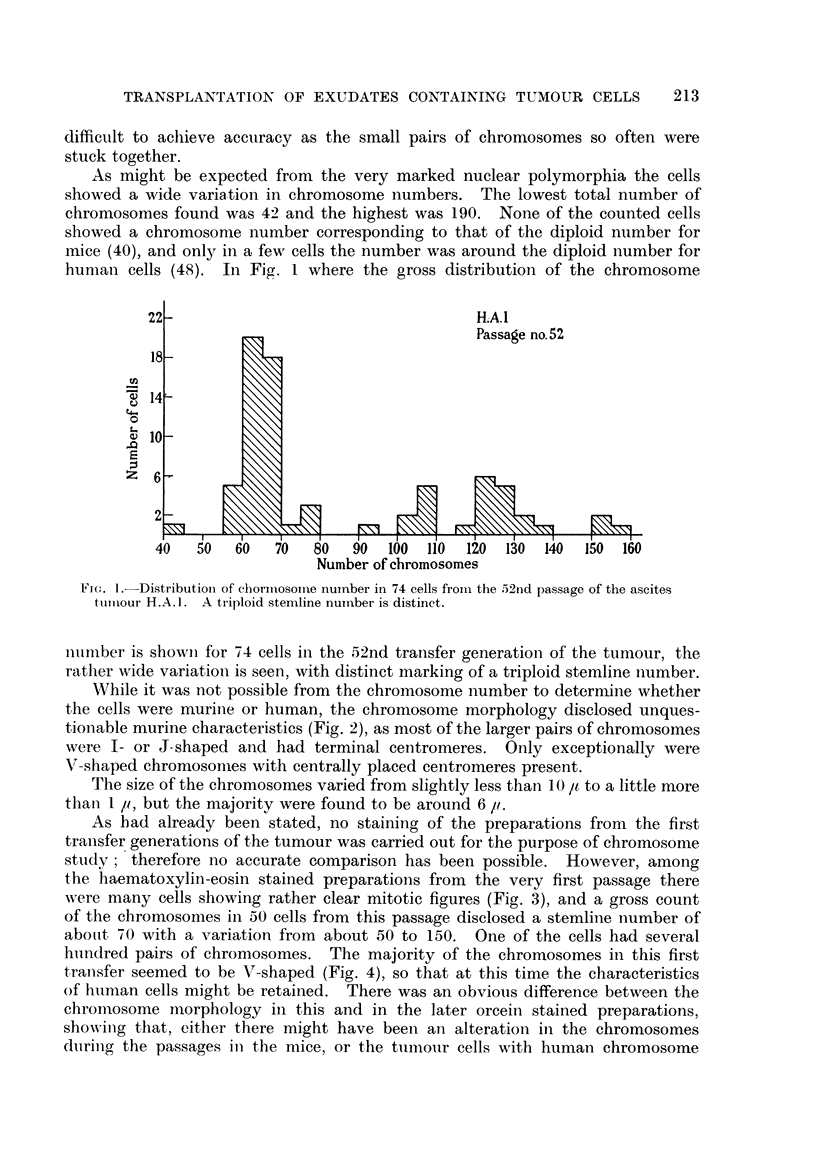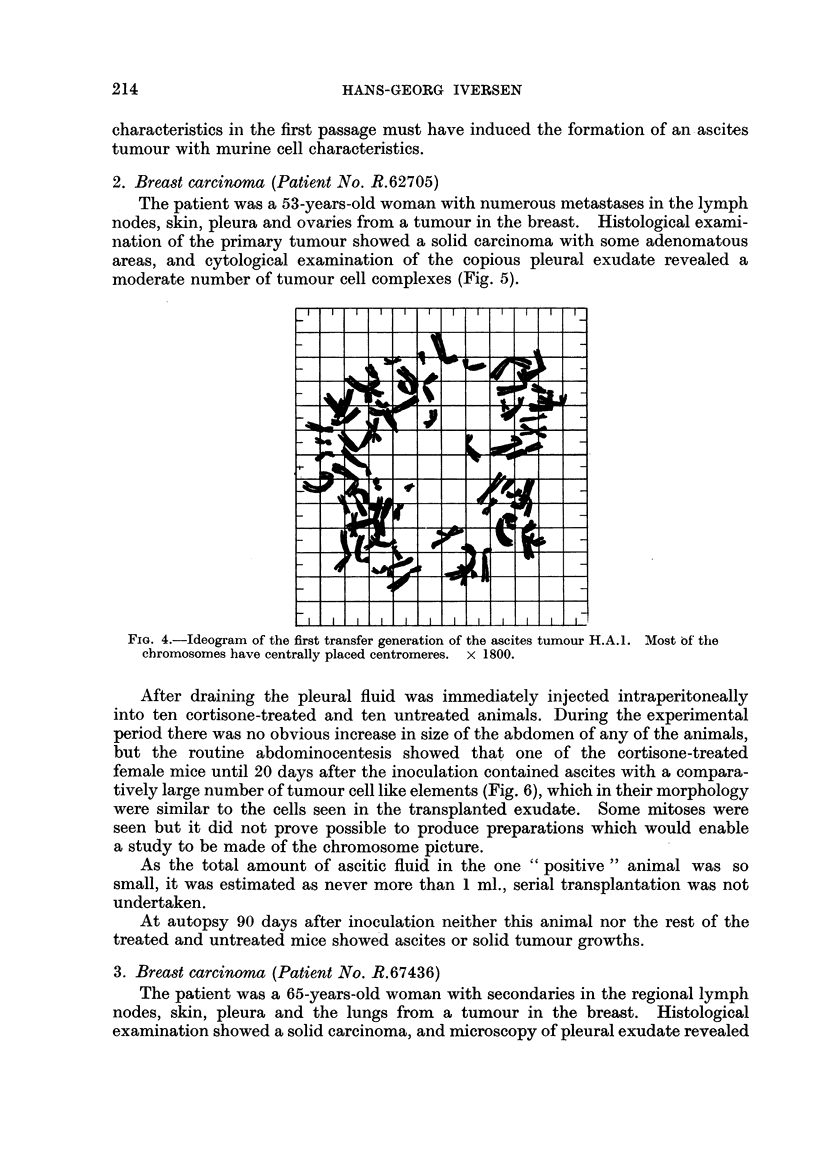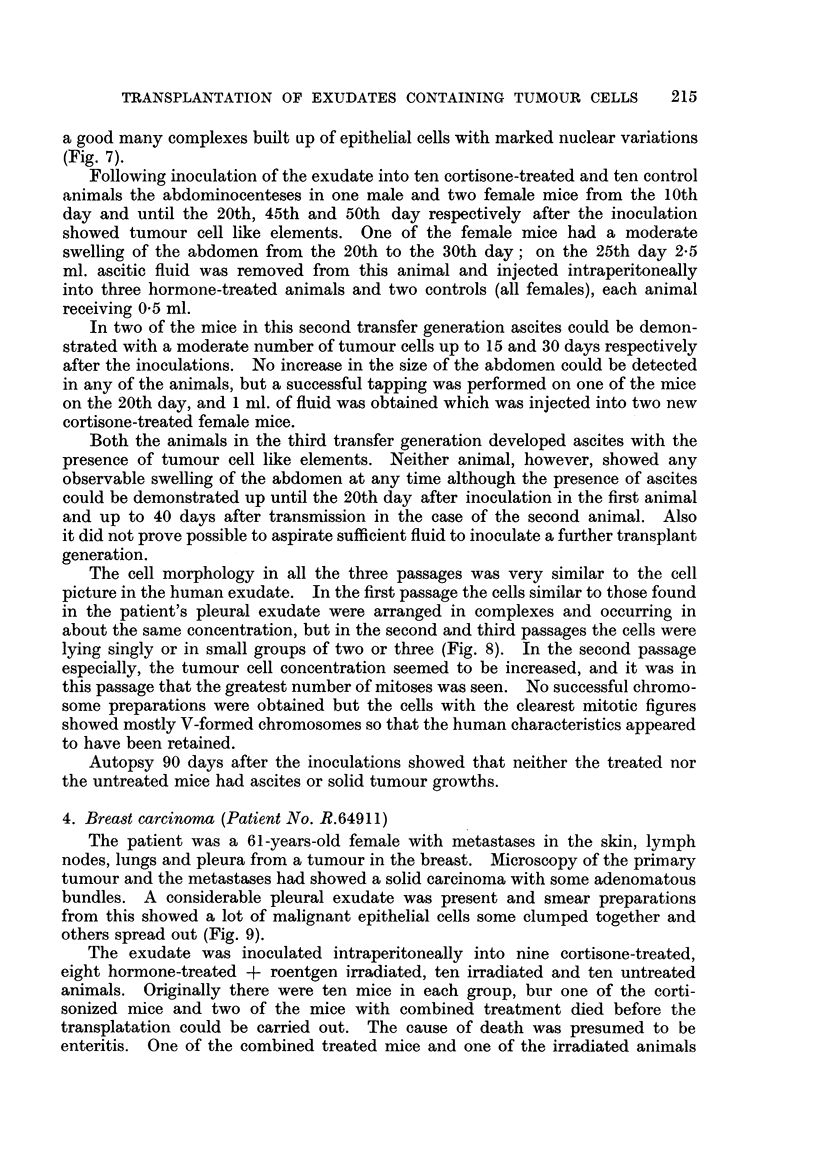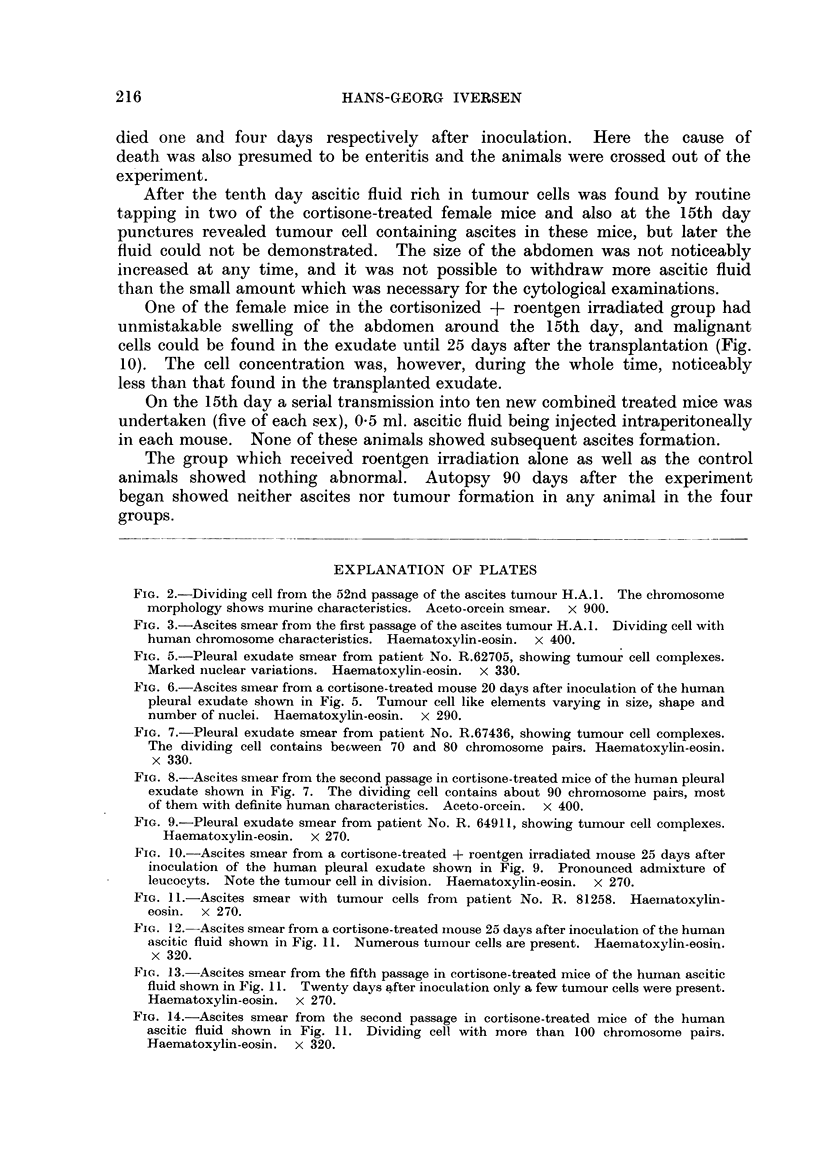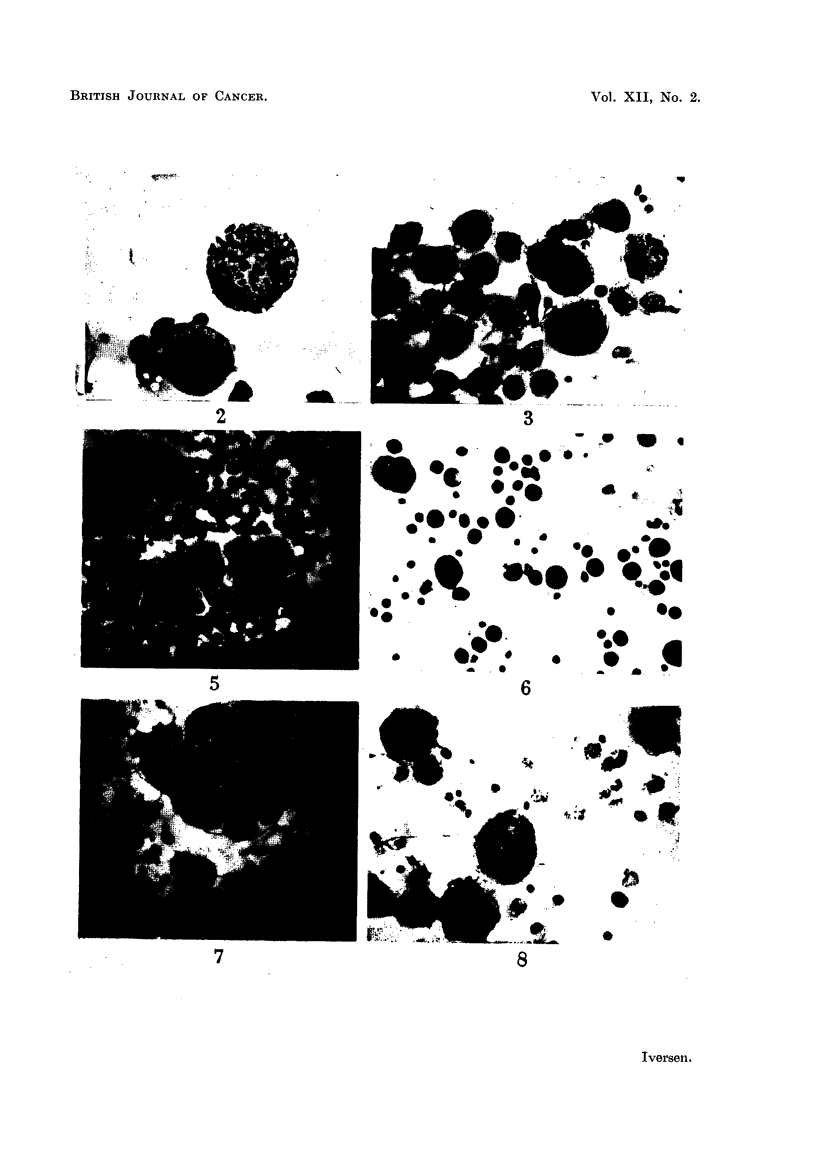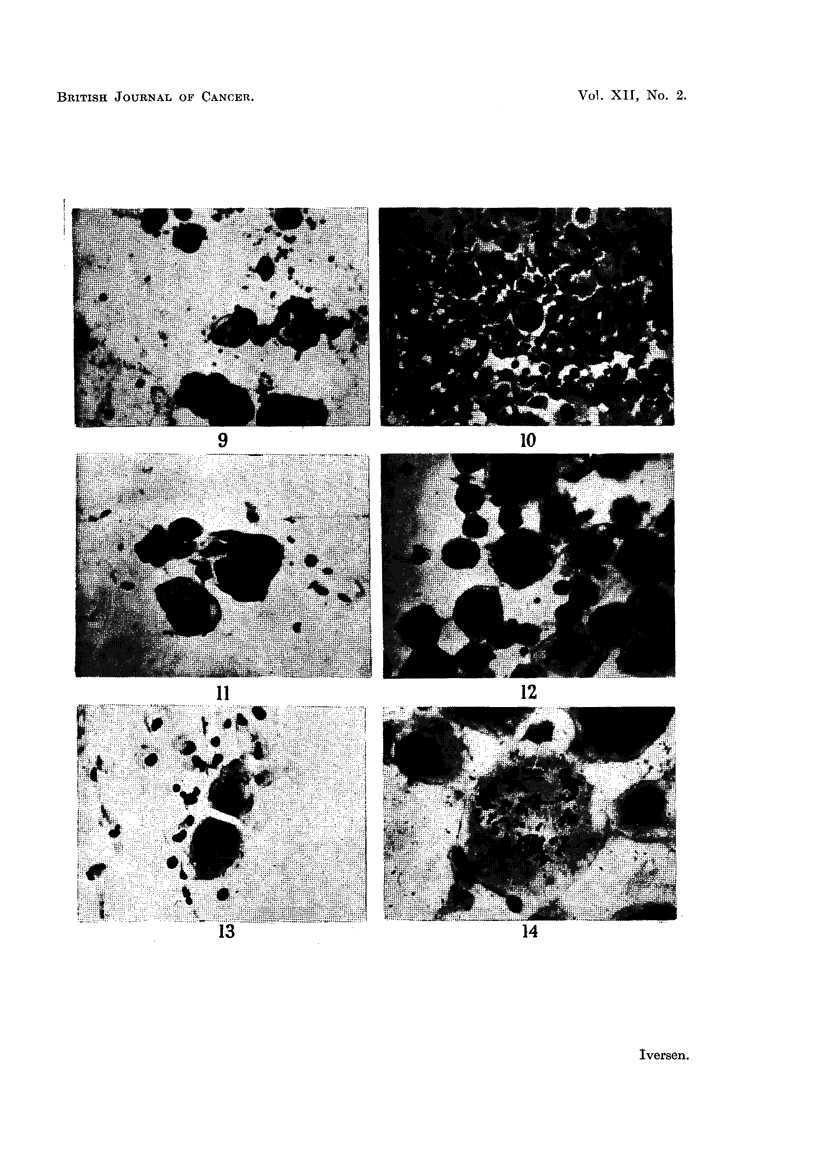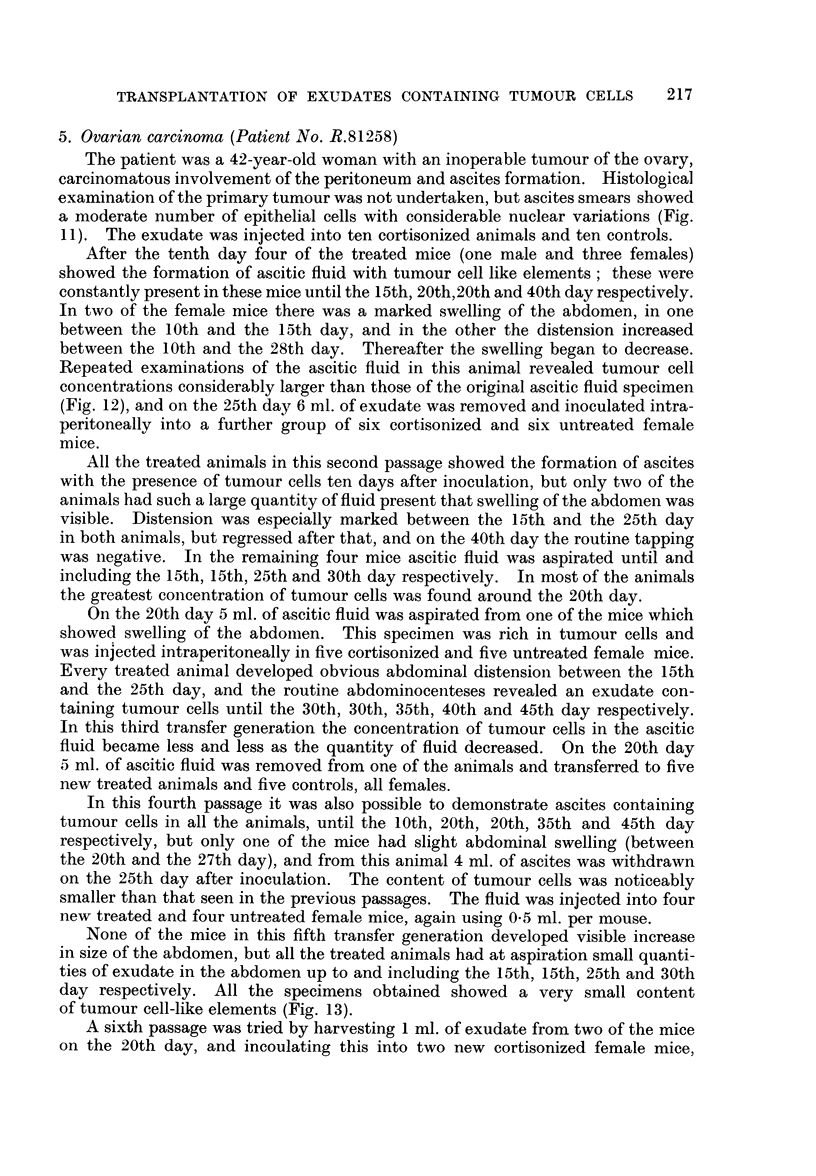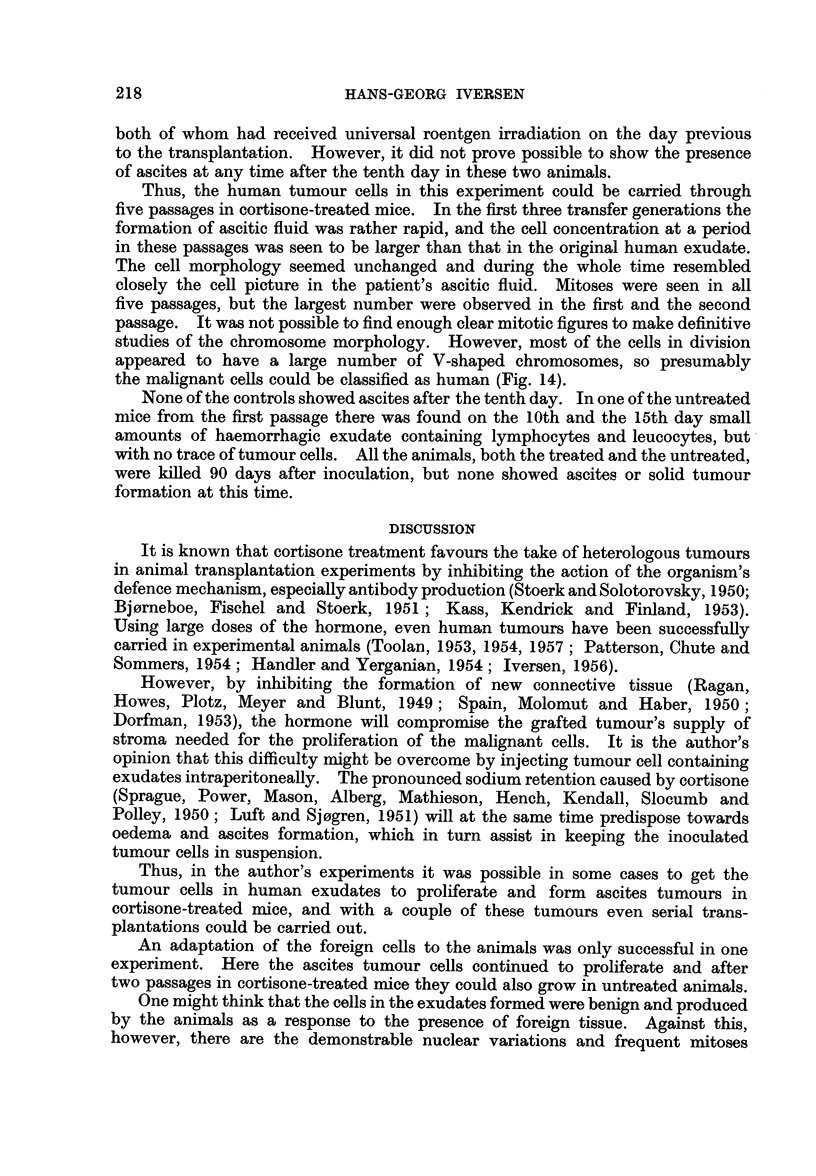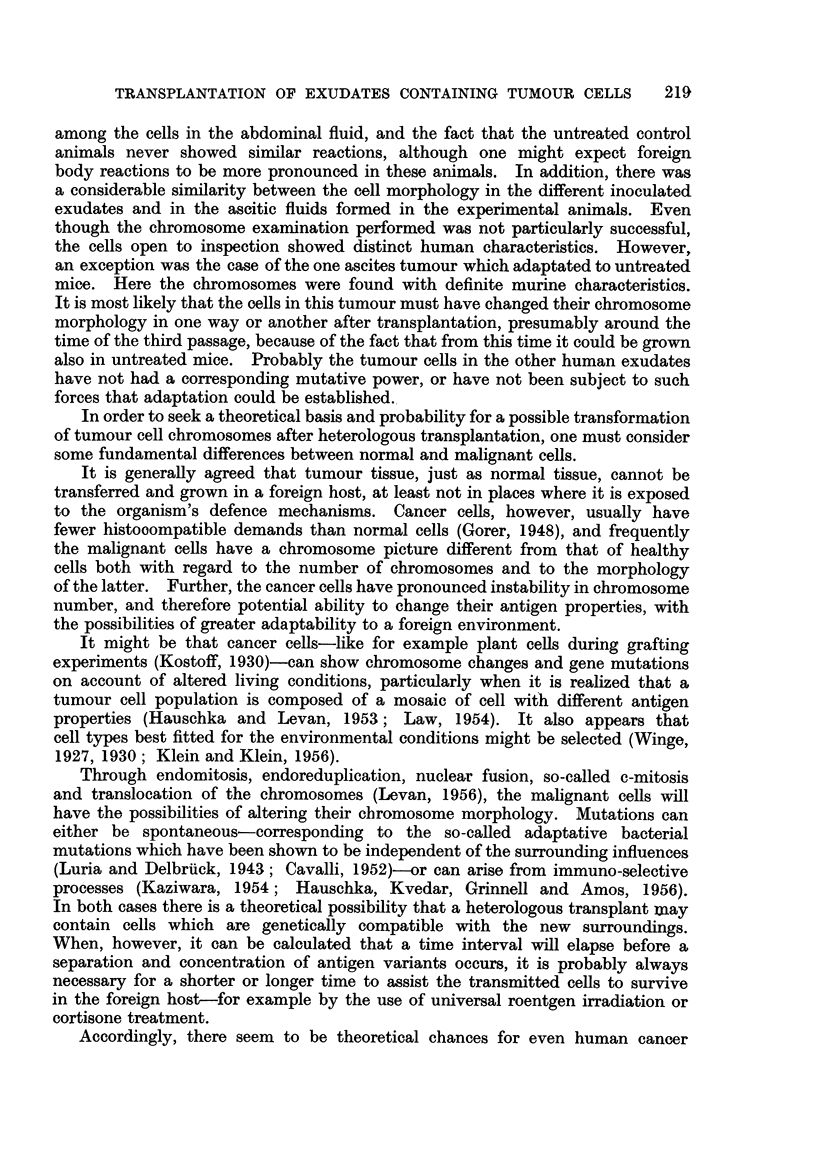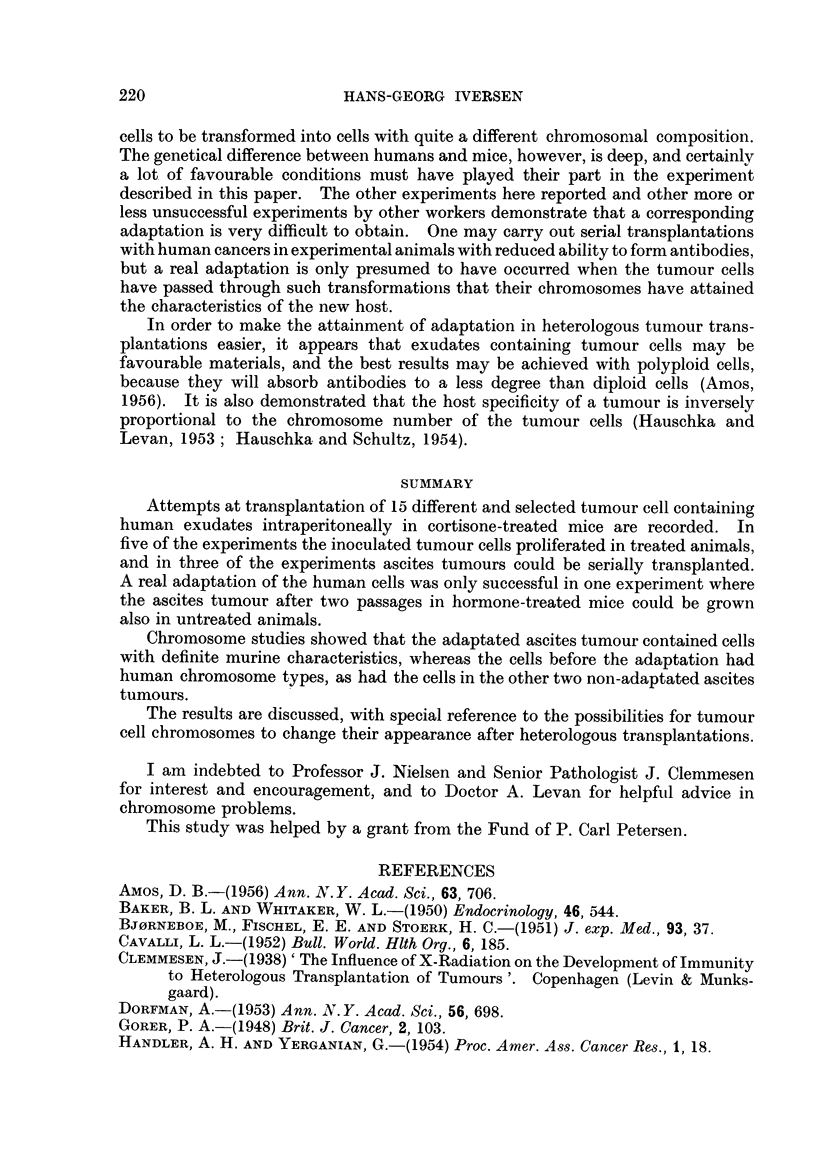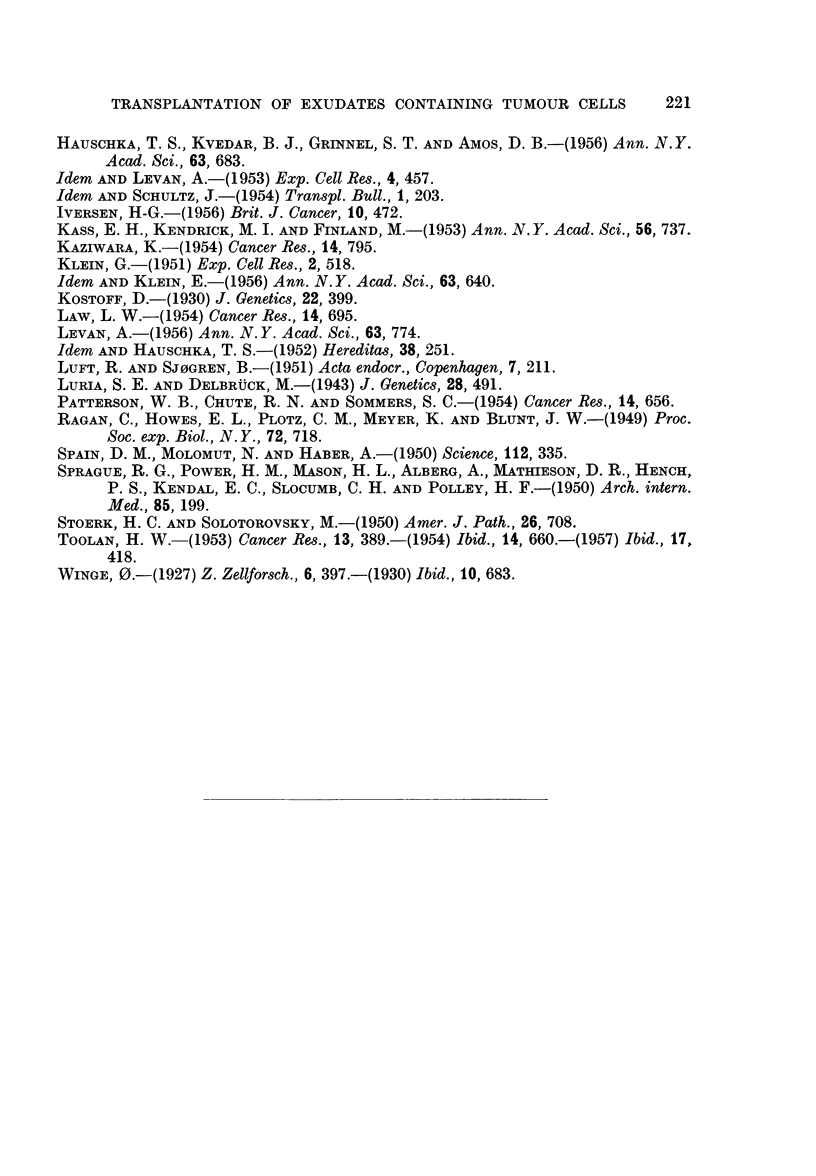# Transplantation from Man to Mouse of Exudates Containing Tumour Cells

**DOI:** 10.1038/bjc.1958.25

**Published:** 1958-06

**Authors:** Hans-Georg Iversen

## Abstract

**Images:**


### TRANSPLANTATION FROM MAN TO MOUSE OF EXUDATES CONTAINING TUMOUR CELLS

HANS-GEORG IVERSEN

*From the Finsen Institute and Radium Centre, Copenhagen*

Received for publication March 4, 1958

IN an earlier article on the transplantation of human tumours into cortisone-treated mice (Iversen, 1956), it was shown that apart from the prolonged survival of several transplanted solid tumours in one or more of the animals in the treated groups, there were found to be especially favourable conditions for implantations when ascitic fluid containing a high percentage of tumour cells was inoculated intraperitoneally in the experimental animals. This procedure resulted in the development of an ascites tumour in four out of nine cortisone-treated mice, and this tumour showed increasing growth rate and ability to adapt to the animals. As early as from the third passage it could be carried also in untreated mice.

One of the great difficulties found when attempting to obtain a successful take of heterologous tumours, is that the proliferating tumour cells must receive a supply of stroma from the animal host (Clemmesen, 1938), and the use of large doses of cortisone for the purpose of depressing the production of antibodies will restrain the formation of fibroblasts (Ragan, Howes, Plotz, Meyer and Blunt, 1949 ; Spain, Molomut and Haber, 1950 ; Baker and Whitaker, 1950), and thus increase the difficulty of obtaining growth of the grafted tumours. By the intraperitoneal transplantation of exudates containing tumour cells it is perhaps possible to overcome this problem, providing the inoculated cells can be kept in suspension. Furthermore, the use of exudates shortens the transplantation time and the intraperitoneal depositing of the malignant cells presumably gives especially favourable growth conditions (Klein, 1951).

The purpose of this present work has therefore been to examine the possibilities for proliferation of tumour cells from different human exudates following intraperitoneal implantation into cortisone-treated and/or roentgen-irradiated mice. Furthermore, an attempt has been made to identify the tumour cell chromosomes, both in the human exudates and in the ascitic fluids formed in the animals, with a view to observing possible changes in the chromosomes if adaptation of the human tumour cells should happen to take place in the animals. The above-mentioned ascites tumour (H.A.1), which until now (February 1958) has passed in more than 120 passages in untreated mice, has also been the object of chromosome examinations and the resulting findings are recorded below.

### MATERIAL AND METHODS

The animals used for the experiments were mice from a subline of the Bagg strain and were two to three months old. Cortisone treatment was commenced four days prior to the transplantations, giving each mouse 1 mg. per day of the preparation " Cortone Merck ". This dose was diluted with physiological saline until 0·1 ml. contained the desired quantity. The injections were given subcutaneously, and were continued every day until the animals died or were killed. The control animals received an injection of 0·1 ml. physiological saline each day. In a few experiments the action of cortisone was supplemented with an universal roentgen irradiation of 200 r 24 hours before the transmission.

Exudates for the implantations were obtained from patients with disseminated malignant diseases, and immediately after aspiration, the fluid was injected intraperitoneally into the animals, giving each mouse 0·5 ml. The patients were selected by preliminary draining and examination of the exudate in smear preparations in order to prove contents of large number of tumour cells or tumour cell complexes, and to ensure cells with marked nuclear polymorphia or mitoses with at least hyperdiploid chromosome number.

Inoculations were carried out alternately on cortisonized and control animals. All the mice were inspected daily and on the tenth day after the transplantation, and on every following fifth day an abdominocentesis was carried out in every animal and present fluid aspirated for cytological examination. Animals still alive 90 days after the transplantation were killed. A complete autopsy was always performed.

Routine, all specimens of aspirated fluid were stained with haematoxolin-eosin. For the examination of the chromosomes an aceto-orcein stained squash preparation was made after the method of Levan and Hauschka (1952), but the aim was only to demonstrate the dominant number of chromosomes in the specimen in question, and by studying the form of the single chromosomes to seek to establish either human or murine characteristics. The author has not felt competent to undertake a more accurate survey of the chromosome morphology. It should be noticed that only the usual haematoxylin-eosin stained preparations were made from the first and most interesting passages of the ascites tumour H.A.1, as it was not fully realized three years ago that a further interesting field might be opened up by a more detailed examination of the chromosomes.

### RESULTS

The results obtained from intraperitoneal implantation of 15 different human exudates containing tumour cells are shown in Table I*a* and I*b*. It was seen that ascites with definite and vigorous tumour cells were found after ten days in one or more of the treated animals in five of these experiments, whereas tumour cells were never found in the untreated animals after this time.

In some of the positive animals from these five series the content of tumour cells was considerably larger than that which had been present in the original exudate transplanted, and not infrequently mitoses were seen.

In a few cases successful transmission of the created ascites tumours into more transfer generations of treated animals was possible, but no success was achieved with the attempt to obtain permanent growths comparable with that seen in the earlier experiment with the H.A.1 tumour.

Where possible a cytological comparison was made between the tumour cells in the transplanted exudate and in the ascites from the animals. This was in order to examine the nature of the chromosomes and an eventual gross change in their morphology.

A description of the experimental conditions in the five " positive " experiments will be given in the following.

TABLE I.—*List of Human Tumour Cell Containing Exudates Inoculated in Mice Treated with Cortisone, Cortisone + Roentgen Irradiation or Roentgen Irradiation Alone*

(The parentheses in the result columns indicate the longest period (in days) after which tumour cells were found in the groups concerned.)

| Patient No. | Sex. | Nature of primary tumour | Nature of exudate used | Number of mice with takes: Cort. | Cort. + Rtg. | Rtg. | Control. |
|---|---|---|---|---|---|---|---|
| | | (a) " *Positive* " *Group. Tumour Cells for* 10 *days or more* | | | | | |
| R.64561 | F. | Squamous cell. carc. (cervix) Solid carcinoma (breast) | Ascitic fluid | 4/9 (Permanent growth) | — | — | 0/10 |
| R.62705 | ,, | Solid and adenomatous carcinoma (breast) | Pleural fluid | 1/10 (20) | — | — | 0/10 |
| R.67436 | ,, | Solid carcinoma (breast) | ,, ,, | 3/10 (3 passages) | — | — | 0/10 |
| R.64911 | ,, | Solid and adenomatous carcinoma (breast) | ,, ,, | 2/9 (15) | 1/7 (25) | 0/9 | 0/10 |
| R.81258 | ,, | Solid carcinoma (ovary) | Ascitic fluid | 4/10 (5 passages) | — | — | 0/10 |
| | | (b) " *Negative* " *Group. Tumour Cells not surviving* 10 *days* | | | | | |
| R.78083 | F. | Adenocarcinoma (ovary) | Ascitic fluid | 0/10 | 0/8 | — | 0/10 |
| E.40273 | ,, | Lymphosarcoma | Pleural fluid | 0/10 | — | — | 0/10 |
| R.83428 | ,, | ,, | ,, ,, | 0/10 | — | — | 0/10 |
| R.82239 | ,, | Solid carcinoma (ovary) | Ascitic fluid | 0/9 | 0/8 | 0/10 | 0/9 |
| Z.2482 | M. | Adenocarcinoma (stomach) | ,, ,, | 0/7 | — | — | 0/9 |
| Z.2129 | ,, | Solid carcinoma (oesophagus) | ,, ,, | 0/10 | 0/6 | 0/9 | 0/10 |
| R.76120 | F. | Solid carcinoma (breast) | Pleural fluid | 0/9 | — | — | 0/10 |
| R.84800 | ,, | Adenocarcinoma (ovary) | Ascitic fluid | 0/10 | — | — | 0/10 |
| R.48962 | M. | Lymphosarcoma | Pleural fluid | 0/8 | — | — | 0/10 |
| R.85159 | F. | Reticulosarcoma | Ascitic fluid | 0/9 | — | — | 0/9 |

### 1. *Uterine and breast carcinomas* (*Patient No. R.*64561)

Ascitic fluid containing tumour cells was removed from a 75-years-old woman with metastases from primary carcinomas both in the uterine cervix and the breast. The patient's history and the results of the transplantation have been described previously (Iversen, 1956). It is interesting that an ascites tumour was formed in four out of nine cortisone-treated animals incoculated with ascitic fluid, and from the third transfer generation the tumour would grow also in the untreated animals and with increasing rate of growth. Until the present (February 1958) the tumour has passed through more than 120 passages.

Chromosome analysis of tumour cells from the 15th, 25th, 38th and 52nd passage respectively showed a rather uniform picture in regard to the average number of chromosomes and their morphology.

In these four transfer generations chromosome counts were undertaken. Each time between 50 and 78 cells in the metaphase were counted but it was difficult to achieve accuracy as the small pairs of chromosomes so often were stuck together.

As might be expected from the very marked nuclear polymorphia the cells showed a wide variation in chromosome numbers. The lowest total number of chromosomes found was 42 and the highest was 190. None of the counted cells showed a chromosome number corresponding to that of the diploid number for mice (40), and only in a few cells the number was around the diploid number for human cells (48). In Fig. 1 where the gross distribution of the chromosome

FIG. 1.—Distribution of chormosome number in 74 cells from the 52nd passage of the ascites tumour H.A.1. A triploid stemline number is distinct.

number is shown for 74 cells in the 52nd transfer generation of the tumour, the rather wide variation is seen, with distinct marking of a triploid stemline number.

While it was not possible from the chromosome number to determine whether the cells were murine or human, the chromosome morphology disclosed unquestionable murine characteristics (Fig. 2), as most of the larger pairs of chromosomes were I- or J-shaped and had terminal centromeres. Only exceptionally were V-shaped chromosomes with centrally placed centromeres present.

The size of the chromosomes varied from slightly less than 10 $\mu$ to a little more than 1 $\mu$, but the majority were found to be around 6 $\mu$.

As had already been stated, no staining of the preparations from the first transfer generations of the tumour was carried out for the purpose of chromosome study ; therefore no accurate comparison has been possible. However, among the haematoxylin-eosin stained preparations from the very first passage there were many cells showing rather clear mitotic figures (Fig. 3), and a gross count of the chromosomes in 50 cells from this passage disclosed a stemline number of about 70 with a variation from about 50 to 150. One of the cells had several hundred pairs of chromosomes. The majority of the chromosomes in this first transfer seemed to be V-shaped (Fig. 4), so that at this time the characteristics of human cells might be retained. There was an obvious difference between the chromosome morphology in this and in the later orcein stained preparations, showing that, either there might have been an alteration in the chromosomes during the passages in the mice, or the tumour cells with human chromosome characteristics in the first passage must have induced the formation of an ascites tumour with murine cell characteristics.

2. *Breast carcinoma* (*Patient No. R.*62705)

The patient was a 53-years-old woman with numerous metastases in the lymph nodes, skin, pleura and ovaries from a tumour in the breast. Histological examination of the primary tumour showed a solid carcinoma with some adenomatous areas, and cytological examination of the copious pleural exudate revealed a moderate number of tumour cell complexes (Fig. 5).

FIG. 4.—Ideogram of the first transfer generation of the ascites tumour H.A.1. Most of the chromosomes have centrally placed centromeres. × 1800.

After draining the pleural fluid was immediately injected intraperitoneally into ten cortisone-treated and ten untreated animals. During the experimental period there was no obvious increase in size of the abdomen of any of the animals, but the routine abdominocentesis showed that one of the cortisone-treated female mice until 20 days after the inoculation contained ascites with a comparatively large number of tumour cell like elements (Fig. 6), which in their morphology were similar to the cells seen in the transplanted exudate. Some mitoses were seen but it did not prove possible to produce preparations which would enable a study to be made of the chromosome picture.

As the total amount of ascitic fluid in the one " positive " animal was so small, it was estimated as never more than 1 ml., serial transplantation was not undertaken.

At autopsy 90 days after inoculation neither this animal nor the rest of the treated and untreated mice showed ascites or solid tumour growths.

3. *Breast carcinoma* (*Patient No. R.*67436)

The patient was a 65-years-old woman with secondaries in the regional lymph nodes, skin, pleura and the lungs from a tumour in the breast. Histological examination showed a solid carcinoma, and microscopy of pleural exudate revealed a good many complexes built up of epithelial cells with marked nuclear variations (Fig. 7).

Following inoculation of the exudate into ten cortisone-treated and ten control animals the abdominocenteses in one male and two female mice from the 10th day and until the 20th, 45th and 50th day respectively after the inoculation showed tumour cell like elements. One of the female mice had a moderate swelling of the abdomen from the 20th to the 30th day ; on the 25th day 2·5 ml. ascitic fluid was removed from this animal and injected intraperitoneally into three hormone-treated animals and two controls (all females), each animal receiving 0·5 ml.

In two of the mice in this second transfer generation ascites could be demonstrated with a moderate number of tumour cells up to 15 and 30 days respectively after the inoculations. No increase in the size of the abdomen could be detected in any of the animals, but a successful tapping was performed on one of the mice on the 20th day, and 1 ml. of fluid was obtained which was injected into two new cortisone-treated female mice.

Both the animals in the third transfer generation developed ascites with the presence of tumour cell like elements. Neither animal, however, showed any observable swelling of the abdomen at any time although the presence of ascites could be demonstrated up until the 20th day after inoculation in the first animal and up to 40 days after transmission in the case of the second animal. Also it did not prove possible to aspirate sufficient fluid to inoculate a further transplant generation.

The cell morphology in all the three passages was very similar to the cell picture in the human exudate. In the first passage the cells similar to those found in the patient's pleural exudate were arranged in complexes and occurring in about the same concentration, but in the second and third passages the cells were lying singly or in small groups of two or three (Fig. 8). In the second passage especially, the tumour cell concentration seemed to be increased, and it was in this passage that the greatest number of mitoses was seen. No successful chromosome preparations were obtained but the cells with the clearest mitotic figures showed mostly V-formed chromosomes so that the human characteristics appeared to have been retained.

Autopsy 90 days after the inoculations showed that neither the treated nor the untreated mice had ascites or solid tumour growths.

4. *Breast carcinoma* (*Patient No. R.*64911)

The patient was a 61-years-old female with metastases in the skin, lymph nodes, lungs and pleura from a tumour in the breast. Microscopy of the primary tumour and the metastases had showed a solid carcinoma with some adenomatous bundles. A considerable pleural exudate was present and smear preparations from this showed a lot of malignant epithelial cells some clumped together and others spread out (Fig. 9).

The exudate was inoculated intraperitoneally into nine cortisone-treated, eight hormone-treated + roentgen irradiated, ten irradiated and ten untreated animals. Originally there were ten mice in each group, bur one of the cortisonized mice and two of the mice with combined treatment died before the transplatation could be carried out. The cause of death was presumed to be enteritis. One of the combined treated mice and one of the irradiated animals died one and four days respectively after inoculation. Here the cause of death was also presumed to be enteritis and the animals were crossed out of the experiment.

After the tenth day ascitic fluid rich in tumour cells was found by routine tapping in two of the cortisone-treated female mice and also at the 15th day punctures revealed tumour cell containing ascites in these mice, but later the fluid could not be demonstrated. The size of the abdomen was not noticeably increased at any time, and it was not possible to withdraw more ascitic fluid than the small amount which was necessary for the cytological examinations.

One of the female mice in the cortisonized + roentgen irradiated group had unmistakable swelling of the abdomen around the 15th day, and malignant cells could be found in the exudate until 25 days after the transplantation (Fig. 10). The cell concentration was, however, during the whole time, noticeably less than that found in the transplanted exudate.

On the 15th day a serial transmission into ten new combined treated mice was undertaken (five of each sex), 0·5 ml. ascitic fluid being injected intraperitoneally in each mouse. None of these animals showed subsequent ascites formation.

The group which received roentgen irradiation alone as well as the control animals showed nothing abnormal. Autopsy 90 days after the experiment began showed neither ascites nor tumour formation in any animal in the four groups.

---

### EXPLANATION OF PLATES

FIG. 2.—Dividing cell from the 52nd passage of the ascites tumour H.A.1. The chromosome morphology shows murine characteristics. Aceto-orcein smear. × 900.

FIG. 3.—Ascites smear from the first passage of the ascites tumour H.A.1. Dividing cell with human chromosome characteristics. Haematoxylin-eosin. × 400.

FIG. 5.—Pleural exudate smear from patient No. R.62705, showing tumour cell complexes. Marked nuclear variations. Haematoxylin-eosin. × 330.

FIG. 6.—Ascites smear from a cortisone-treated mouse 20 days after inoculation of the human pleural exudate shown in Fig. 5. Tumour cell like elements varying in size, shape and number of nuclei. Haematoxylin-eosin. × 290.

FIG. 7.—Pleural exudate smear from patient No. R.67436, showing tumour cell complexes. The dividing cell contains between 70 and 80 chromosome pairs. Haematoxylin-eosin. × 330.

FIG. 8.—Ascites smear from the second passage in cortisone-treated mice of the human pleural exudate shown in Fig. 7. The dividing cell contains about 90 chromosome pairs, most of them with definite human characteristics. Aceto-orcein. × 400.

FIG. 9.—Pleural exudate smear from patient No. R. 64911, showing tumour cell complexes. Haematoxylin-eosin. × 270.

FIG. 10.—Ascites smear from a cortisone-treated + roentgen irradiated mouse 25 days after inoculation of the human pleural exudate shown in Fig. 9. Pronounced admixture of leucocyts. Note the tumour cell in division. Haematoxylin-eosin. × 270.

FIG. 11.—Ascites smear with tumour cells from patient No. R. 81258. Haematoxylin-eosin. × 270.

FIG. 12.—Ascites smear from a cortisone-treated mouse 25 days after inoculation of the human ascitic fluid shown in Fig. 11. Numerous tumour cells are present. Haematoxylin-eosin. × 320.

FIG. 13.—Ascites smear from the fifth passage in cortisone-treated mice of the human ascitic fluid shown in Fig. 11. Twenty days after inoculation only a few tumour cells were present. Haematoxylin-eosin. × 270.

FIG. 14.—Ascites smear from the second passage in cortisone-treated mice of the human ascitic fluid shown in Fig. 11. Dividing cell with more than 100 chromosome pairs. Haematoxylin-eosin. × 320.

2

3

5

6

7

8

Iversen.

5. *Ovarian carcinoma* (*Patient No. R.*81258)

The patient was a 42-year-old woman with an inoperable tumour of the ovary, carcinomatous involvement of the peritoneum and ascites formation. Histological examination of the primary tumour was not undertaken, but ascites smears showed a moderate number of epithelial cells with considerable nuclear variations (Fig. 11). The exudate was injected into ten cortisonized animals and ten controls.

After the tenth day four of the treated mice (one male and three females) showed the formation of ascitic fluid with tumour cell like elements ; these were constantly present in these mice until the 15th, 20th,20th and 40th day respectively. In two of the female mice there was a marked swelling of the abdomen, in one between the 10th and the 15th day, and in the other the distension increased between the 10th and the 28th day. Thereafter the swelling began to decrease. Repeated examinations of the ascitic fluid in this animal revealed tumour cell concentrations considerably larger than those of the original ascitic fluid specimen (Fig. 12), and on the 25th day 6 ml. of exudate was removed and inoculated intraperitoneally into a further group of six cortisonized and six untreated female mice.

All the treated animals in this second passage showed the formation of ascites with the presence of tumour cells ten days after inoculation, but only two of the animals had such a large quantity of fluid present that swelling of the abdomen was visible. Distension was especially marked between the 15th and the 25th day in both animals, but regressed after that, and on the 40th day the routine tapping was negative. In the remaining four mice ascitic fluid was aspirated until and including the 15th, 15th, 25th and 30th day respectively. In most of the animals the greatest concentration of tumour cells was found around the 20th day.

On the 20th day 5 ml. of ascitic fluid was aspirated from one of the mice which showed swelling of the abdomen. This specimen was rich in tumour cells and was injected intraperitoneally in five cortisonized and five untreated female mice. Every treated animal developed obvious abdominal distension between the 15th and the 25th day, and the routine abdominocenteses revealed an exudate containing tumour cells until the 30th, 30th, 35th, 40th and 45th day respectively. In this third transfer generation the concentration of tumour cells in the ascitic fluid became less and less as the quantity of fluid decreased. On the 20th day 5 ml. of ascitic fluid was removed from one of the animals and transferred to five new treated animals and five controls, all females.

In this fourth passage it was also possible to demonstrate ascites containing tumour cells in all the animals, until the 10th, 20th, 20th, 35th and 45th day respectively, but only one of the mice had slight abdominal swelling (between the 20th and the 27th day), and from this animal 4 ml. of ascites was withdrawn on the 25th day after inoculation. The content of tumour cells was noticeably smaller than that seen in the previous passages. The fluid was injected into four new treated and four untreated female mice, again using 0·5 ml. per mouse.

None of the mice in this fifth transfer generation developed visible increase in size of the abdomen, but all the treated animals had at aspiration small quantities of exudate in the abdomen up to and including the 15th, 15th, 25th and 30th day respectively. All the specimens obtained showed a very small content of tumour cell-like elements (Fig. 13).

A sixth passage was tried by harvesting 1 ml. of exudate from two of the mice on the 20th day, and incoulating this into two new cortisonized female mice, both of whom had received universal roentgen irradiation on the day previous to the transplantation. However, it did not prove possible to show the presence of ascites at any time after the tenth day in these two animals.

Thus, the human tumour cells in this experiment could be carried through five passages in cortisone-treated mice. In the first three transfer generations the formation of ascitic fluid was rather rapid, and the cell concentration at a period in these passages was seen to be larger than that in the original human exudate. The cell morphology seemed unchanged and during the whole time resembled closely the cell picture in the patient's ascitic fluid. Mitoses were seen in all five passages, but the largest number were observed in the first and the second passage. It was not possible to find enough clear mitotic figures to make definitive studies of the chromosome morphology. However, most of the cells in division appeared to have a large number of V-shaped chromosomes, so presumably the malignant cells could be classified as human (Fig. 14).

None of the controls showed ascites after the tenth day. In one of the untreated mice from the first passage there was found on the 10th and the 15th day small amounts of haemorrhagic exudate containing lymphocytes and leucocytes, but with no trace of tumour cells. All the animals, both the treated and the untreated, were killed 90 days after inoculation, but none showed ascites or solid tumour formation at this time.

### DISCUSSION

It is known that cortisone treatment favours the take of heterologous tumours in animal transplantation experiments by inhibiting the action of the organism's defence mechanism, especially antibody production (Stoerk and Solotorovsky, 1950; Bjørneboe, Fischel and Stoerk, 1951 ; Kass, Kendrick and Finland, 1953). Using large doses of the hormone, even human tumours have been successfully carried in experimental animals (Toolan, 1953, 1954, 1957 ; Patterson, Chute and Sommers, 1954 ; Handler and Yerganian, 1954 ; Iversen, 1956).

However, by inhibiting the formation of new connective tissue (Ragan, Howes, Plotz, Meyer and Blunt, 1949 ; Spain, Molomut and Haber, 1950 ; Dorfman, 1953), the hormone will compromise the grafted tumour's supply of stroma needed for the proliferation of the malignant cells. It is the author's opinion that this difficulty might be overcome by injecting tumour cell containing exudates intraperitoneally. The pronounced sodium retention caused by cortisone (Sprague, Power, Mason, Alberg, Mathieson, Hench, Kendall, Slocumb and Polley, 1950 ; Luft and Sjøgren, 1951) will at the same time predispose towards oedema and ascites formation, which in turn assist in keeping the inoculated tumour cells in suspension.

Thus, in the author's experiments it was possible in some cases to get the tumour cells in human exudates to proliferate and form ascites tumours in cortisone-treated mice, and with a couple of these tumours even serial transplantations could be carried out.

An adaptation of the foreign cells to the animals was only successful in one experiment. Here the ascites tumour cells continued to proliferate and after two passages in cortisone-treated mice they could also grow in untreated animals.

One might think that the cells in the exudates formed were benign and produced by the animals as a response to the presence of foreign tissue. Against this, however, there are the demonstrable nuclear variations and frequent mitoses among the cells in the abdominal fluid, and the fact that the untreated control animals never showed similar reactions, although one might expect foreign body reactions to be more pronounced in these animals. In addition, there was a considerable similarity between the cell morphology in the different inoculated exudates and in the ascitic fluids formed in the experimental animals. Even though the chromosome examination performed was not particularly successful, the cells open to inspection showed distinct human characteristics. However, an exception was the case of the one ascites tumour which adaptated to untreated mice. Here the chromosomes were found with definite murine characteristics. It is most likely that the cells in this tumour must have changed their chromosome morphology in one way or another after transplantation, presumably around the time of the third passage, because of the fact that from this time it could be grown also in untreated mice. Probably the tumour cells in the other human exudates have not had a corresponding mutative power, or have not been subject to such forces that adaptation could be established.

In order to seek a theoretical basis and probability for a possible transformation of tumour cell chromosomes after heterologous transplantation, one must consider some fundamental differences between normal and malignant cells.

It is generally agreed that tumour tissue, just as normal tissue, cannot be transferred and grown in a foreign host, at least not in places where it is exposed to the organism's defence mechanisms. Cancer cells, however, usually have fewer histocompatible demands than normal cells (Gorer, 1948), and frequently the malignant cells have a chromosome picture different from that of healthy cells both with regard to the number of chromosomes and to the morphology of the latter. Further, the cancer cells have pronounced instability in chromosome number, and therefore potential ability to change their antigen properties, with the possibilities of greater adaptability to a foreign environment.

It might be that cancer cells—like for example plant cells during grafting experiments (Kostoff, 1930)—can show chromosome changes and gene mutations on account of altered living conditions, particularly when it is realized that a tumour cell population is composed of a mosaic of cell with different antigen properties (Hauschka and Levan, 1953; Law, 1954). It also appears that cell types best fitted for the environmental conditions might be selected (Winge, 1927, 1930; Klein and Klein, 1956).

Through endomitosis, endoreduplication, nuclear fusion, so-called c-mitosis and translocation of the chromosomes (Levan, 1956), the malignant cells will have the possibilities of altering their chromosome morphology. Mutations can either be spontaneous—corresponding to the so-called adaptative bacterial mutations which have been shown to be independent of the surrounding influences (Luria and Delbrück, 1943; Cavalli, 1952)—or can arise from immuno-selective processes (Kaziwara, 1954; Hauschka, Kvedar, Grinnell and Amos, 1956). In both cases there is a theoretical possibility that a heterologous transplant may contain cells which are genetically compatible with the new surroundings. When, however, it can be calculated that a time interval will elapse before a separation and concentration of antigen variants occurs, it is probably always necessary for a shorter or longer time to assist the transmitted cells to survive in the foreign host—for example by the use of universal roentgen irradiation or cortisone treatment.

Accordingly, there seem to be theoretical chances for even human cancer cells to be transformed into cells with quite a different chromosomal composition. The genetical difference between humans and mice, however, is deep, and certainly a lot of favourable conditions must have played their part in the experiment described in this paper. The other experiments here reported and other more or less unsuccessful experiments by other workers demonstrate that a corresponding adaptation is very difficult to obtain. One may carry out serial transplantations with human cancers in experimental animals with reduced ability to form antibodies, but a real adaptation is only presumed to have occurred when the tumour cells have passed through such transformations that their chromosomes have attained the characteristics of the new host.

In order to make the attainment of adaptation in heterologous tumour transplantations easier, it appears that exudates containing tumour cells may be favourable materials, and the best results may be achieved with polyploid cells, because they will absorb antibodies to a less degree than diploid cells (Amos, 1956). It is also demonstrated that the host specificity of a tumour is inversely proportional to the chromosome number of the tumour cells (Hauschka and Levan, 1953 ; Hauschka and Schultz, 1954).

### SUMMARY

Attempts at transplantation of 15 different and selected tumour cell containing human exudates intraperitoneally in cortisone-treated mice are recorded. In five of the experiments the inoculated tumour cells proliferated in treated animals, and in three of the experiments ascites tumours could be serially transplanted. A real adaptation of the human cells was only successful in one experiment where the ascites tumour after two passages in hormone-treated mice could be grown also in untreated animals.

Chromosome studies showed that the adaptated ascites tumour contained cells with definite murine characteristics, whereas the cells before the adaptation had human chromosome types, as had the cells in the other two non-adaptated ascites tumours.

The results are discussed, with special reference to the possibilities for tumour cell chromosomes to change their appearance after heterologous transplantations.

I am indebted to Professor J. Nielsen and Senior Pathologist J. Clemmesen for interest and encouragement, and to Doctor A. Levan for helpful advice in chromosome problems.

This study was helped by a grant from the Fund of P. Carl Petersen.

### REFERENCES

Amos, D. B.—(1956) *Ann. N.Y. Acad. Sci.*, **63**, 706.
Baker, B. L. and Whitaker, W. L.—(1950) *Endocrinology*, **46**, 544.
Bjørneboe, M., Fischel, E. E. and Stoerk, H. C.—(1951) *J. exp. Med.*, **93**, 37.
Cavalli, L. L.—(1952) *Bull. World. Hlth Org.*, **6**, 185.
Clemmesen, J.—(1938) ‘ The Influence of X-Radiation on the Development of Immunity to Heterologous Transplantation of Tumours ’. Copenhagen (Levin & Munksgaard).
Dorfman, A.—(1953) *Ann. N.Y. Acad. Sci.*, **56**, 698.
Gorer, P. A.—(1948) *Brit. J. Cancer*, **2**, 103.
Handler, A. H. and Yerganian, G.—(1954) *Proc. Amer. Ass. Cancer Res.*, **1**, 18.

HAUSCHKA, T. S., KVEDAR, B. J., GRINNEL, S. T. AND AMOS, D. B.—(1956) *Ann. N.Y. Acad. Sci.*, **63**, 683.
*Idem* AND LEVAN, A.—(1953) *Exp. Cell Res.*, **4**, 457.
*Idem* AND SCHULTZ, J.—(1954) *Transpl. Bull.*, **1**, 203.
IVERSEN, H-G.—(1956) *Brit. J. Cancer*, **10**, 472.
KASS, E. H., KENDRICK, M. I. AND FINLAND, M.—(1953) *Ann. N.Y. Acad. Sci.*, **56**, 737.
KAZIWARA, K.—(1954) *Cancer Res.*, **14**, 795.
KLEIN, G.—(1951) *Exp. Cell Res.*, **2**, 518.
*Idem* AND KLEIN, E.—(1956) *Ann. N.Y. Acad. Sci.*, **63**, 640.
KOSTOFF, D.—(1930) *J. Genetics*, **22**, 399.
LAW, L. W.—(1954) *Cancer Res.*, **14**, 695.
LEVAN, A.—(1956) *Ann. N.Y. Acad. Sci.*, **63**, 774.
*Idem* AND HAUSCHKA, T. S.—(1952) *Hereditas*, **38**, 251.
LUFT, R. AND SJØGREN, B.—(1951) *Acta endocr., Copenhagen*, **7**, 211.
LURIA, S. E. AND DELBRÜCK, M.—(1943) *J. Genetics*, **28**, 491.
PATTERSON, W. B., CHUTE, R. N. AND SOMMERS, S. C.—(1954) *Cancer Res.*, **14**, 656.
RAGAN, C., HOWES, E. L., PLOTZ, C. M., MEYER, K. AND BLUNT, J. W.—(1949) *Proc. Soc. exp. Biol., N.Y.*, **72**, 718.
SPAIN, D. M., MOLOMUT, N. AND HABER, A.—(1950) *Science*, **112**, 335.
SPRAGUE, R. G., POWER, H. M., MASON, H. L., ALBERG, A., MATHIESON, D. R., HENCH, P. S., KENDAL, E. C., SLOCUMB, C. H. AND POLLEY, H. F.—(1950) *Arch. intern. Med.*, **85**, 199.
STOERK, H. C. AND SOLOTOROVSKY, M.—(1950) *Amer. J. Path.*, **26**, 708.
TOOLAN, H. W.—(1953) *Cancer Res.*, **13**, 389.—(1954) *Ibid.*, **14**, 660.—(1957) *Ibid.*, **17**, 418.
WINGE, Ø.—(1927) *Z. Zellforsch.*, **6**, 397.—(1930) *Ibid.*, **10**, 683.